# Editorial: Pharmacological approaches targeting neutrophilic inflammation: Volume II

**DOI:** 10.3389/fphar.2022.1084026

**Published:** 2022-11-22

**Authors:** Alexey V. Sokolov, Boris V. Chernyak, Roman A. Zinovkin, Tsong-Long Hwang, Galina F. Sud’ina

**Affiliations:** ^1^ Institute of Experimental Medicine, St. Petersburg, Russia; ^2^ Belozersky Institute of Physico-Chemical Biology, Lomonosov Moscow State University, Moscow, Russia; ^3^ The “Russian Clinical Research Center for Gerontology” of the Ministry of Healthcare of the Russian Federation, Pirogov Russian National Research Medical University, Moscow, Russia; ^4^ Graduate Institute of Natural Products, Chang Gung University, Taoyuan, Taiwan; ^5^ Graduate Institute of Health Industry Technology, Chang Gung University of Science and Technology, Taoyuan, Taiwan; ^6^ Department of Anesthesiology, Chang Gung Memorial Hospital, Taoyuan, Taiwan

**Keywords:** neutrophils, inflammation, acute lung injury, sepsis, neutrophil extracellular traps (NETs), phagocytosis

Neutrophils are essential for maintaining homeostasis and the functioning of the innate immune system. Neutrophils are the first immune cells to respond, and they release numerous types of substances that are crucial for eliminating microbes. However, it can also result in collateral tissue damage if neutrophilic activity is overdone. Thus, regulation of neutrophilic activity is of great importance for the treatment of many pathological conditions. The Research Topic aims to highlight the ongoing advancement in the pharmacological approaches targeting neutrophilic inflammation.

Many acute and chronic lung disorders are accompanied by increased neutrophilic infiltration. Acute lung injury (ALI) and acute respiratory distress syndrome (ARDS) are the main causes of acute respiratory failure in seriously ill patients, with critical role of neutrophils in epithelial and endothelial dysfunction. It is not surprising that nearly half of the papers on this Research Topic are devoted to inflammatory diseases of the respiratory tract.

Earlier studies has demonstrated increased levels of the pro-fibrotic, ß-galactoside-binding lectin Galectin-3, which is involved in neutrophils recruitment and stimulation, increased in the lungs during ALI (Humphries et al., 2021). It has been suggested that Galectin-3 inhibition may be a promising therapeutic approach in the treatment of ALI. In this Research Topic, the same research group by Humphries et al. reported that the Galectin-3 inhibitor GB0139 reduced inflammation and decreased neutrophil activation in an ALI model. GB0139 inhibited neutrophil recruitment in LPS-induced lung inflammation while accelerating neutrophil apoptosis. The study supports the development of Galectin-3 inhibitors as a therapeutic agent for the treatment of ALI.

In asthma, airways neutrophils recruitment and neutrophil extracellular traps (NETs) formation are associated with disease severity (Janssen et al., 2022), and neutrophilic asthma is poorly controlled by conventional therapy. Kim et al. have explored targeting of extracellular traps formed by host DNA of leukocyte origin to inhibit inflammatory asthma. The authors found that microRNA (miR)-155 regulated the release of extracellular traps. The level of miR-155 was increased in asthma and the inhibition of miR-155 mitigated neutrophilic asthma.

The pharmacological approach to ALI treatment by classical Chinese medicine Qing-Jin-Hua-Tang-Decoction (QJHTD) was conducted by Xiao et al. Network pharmacology with experimental validation identified the active components, effective targets and potential mechanisms of action of QJHTD in ALI. Some components prevented thrombosis in ALI. Direct binding to thrombin and inhibition of its activity in micromolar range was evidenced for baicalein, wogonin, and baicalin. Interestingly, QJHTD also inhibited NETs formation. The formation of NETs containing chromatin makes a significant contribution to antimicrobial protection but also to the pathogenesis of various inflammatory diseases. In particular, excessive NETs formation has been shown to play an important role in ALI and coronavirus disease 2019 (COVID-19) (Cesta et al., 2021) (Scozzi et al., 2022).

Hyperinflammation in COVID-19 is characterized by elevated blood levels of neutrophils and neutrophil activation, accompanied by hypercoagulability and blood clotting, which is the main cause of death in this disease. The urgent task is the search for possible approaches to reduce the pro-inflammatory functions of neutrophils while preserving their protective functions. Masso-Silva et al. have found, that intravenous immunoglobulin (IVIG) reduced neutrophil inflammatory pathways in patients with COVID-19. IVIG was shown to dose-dependently inhibit NETs production and oxidative burst but did not affect *ex vivo* neutrophil phagocytosis. Plasma levels of both extracellular DNA and neutrophil elastase in patients with COVID-19 were significantly lower after IVIG treatment. These findings present a new perspective for the application of neutrophil modulators to the therapeutic repertoire of COVID-19.

In severe COVID-19, neutrophils play an important role in the pathogenesis of ARDS, vascular disease, and sepsis (Ventura-Santana et al., 2022). Huang et al. presented an interesting report on the protective effects of d-tagatose in ARDS model induced by oleic acid in rats, as a basis for the development of new therapeutic approaches. d-tagatose improved oxygenation function, reduced respiratory acidosis, improved vascular permeability, and maintained the stability of the alveolar structure.

Chronic viral infections induce sustained inflammatory cytokine signaling and oxidative stress that are associated with atherogenesis. However, the impact of hyperinflammation in COVID-19 on atherogenesis remains unclear. The study by Nie et al. showed that circulating endotoxin levels and intestinal neutrophil elastase activity positively correlated with the progression of atherosclerosis in patients. The selective neutrophil elastase inhibitor sivelestat reduced intestinal permeability and endotoxemia in Apo E−/− atherosclerotic mice. In conclusion, application of sivelestat was proposed as a promising approach to the treatment of atherosclerosis and the protection of intestinal homeostasis, which plays a critical role in pathogenesis of atherosclerosis.

Neonatal neutrophils are less sensitive to many stimuli, making children more susceptible to sepsis than adults (Fleischmann-Struzek et al., 2018). The role of checkpoint inhibitor proteins in the immune response to sepsis in both adults and neonates is reviewed by Hensler et al. The authors point out to significant gaps in the management of neonatal sepsis and suggest that checkpoint inhibitor proteins such as PD-1, PD-L1, VISTA, and HVEM may be useful for the diagnosis and treatment of patients with sepsis.

Neutrophils are involved in defense mechanisms against microbial pathogens *via* phagocytosis and ROS production. At the same time, neutrophils may also be involved in the pathogenesis of some infections by inducing oxidative stress, releasing toxic granules and NETs. The role of neutrophils in malaria infection has been reviewed by Babatunde and Adenuga. The malaria parasite is known to inhibit the antimicrobial functions of neutrophils, making malaria patients more susceptible to secondary opportunistic *Salmonella* infections. Hemolysis of red blood cells in malaria is responsible for the inhibition of phagocytosis, ROS production and neutrophil migration. The authors discussed some conflicting data on the use of murine models to study the role of neutrophils in malaria.

Bacterial infections remain the leading cause of death, and pharmacological approaches to enhance phagocytosis are in high demand. Jacob S. Brenner and colleagues recently published the study identifying the properties of nanoparticle leading to neutrophil tropism in inflamed lungs (Myerson et al., 2022). In continuation to these studies, Rubey et al. developed an approach to enhance bacterial neutrophil phagocytosis. The authors studied a wide class of neutrophil-tropic nanoparticles and demonstrated that they enhance phagocytosis and enter the same sites as bacteria inside neutrophils. It has been suggested that these nanoparticles can serve as useful drug carriers to alleviate bacterial diseases.

The regulatory role of microRNAs (miRNAs) in phagocytosis has been reviewed by Wang et al. The authors noticed that the effects of miRNAs on neutrophils and macrophages are highly environmentally dependent and formulated further steps to determine the therapeutic utility of miRNAs.

**SCHEME 1:**
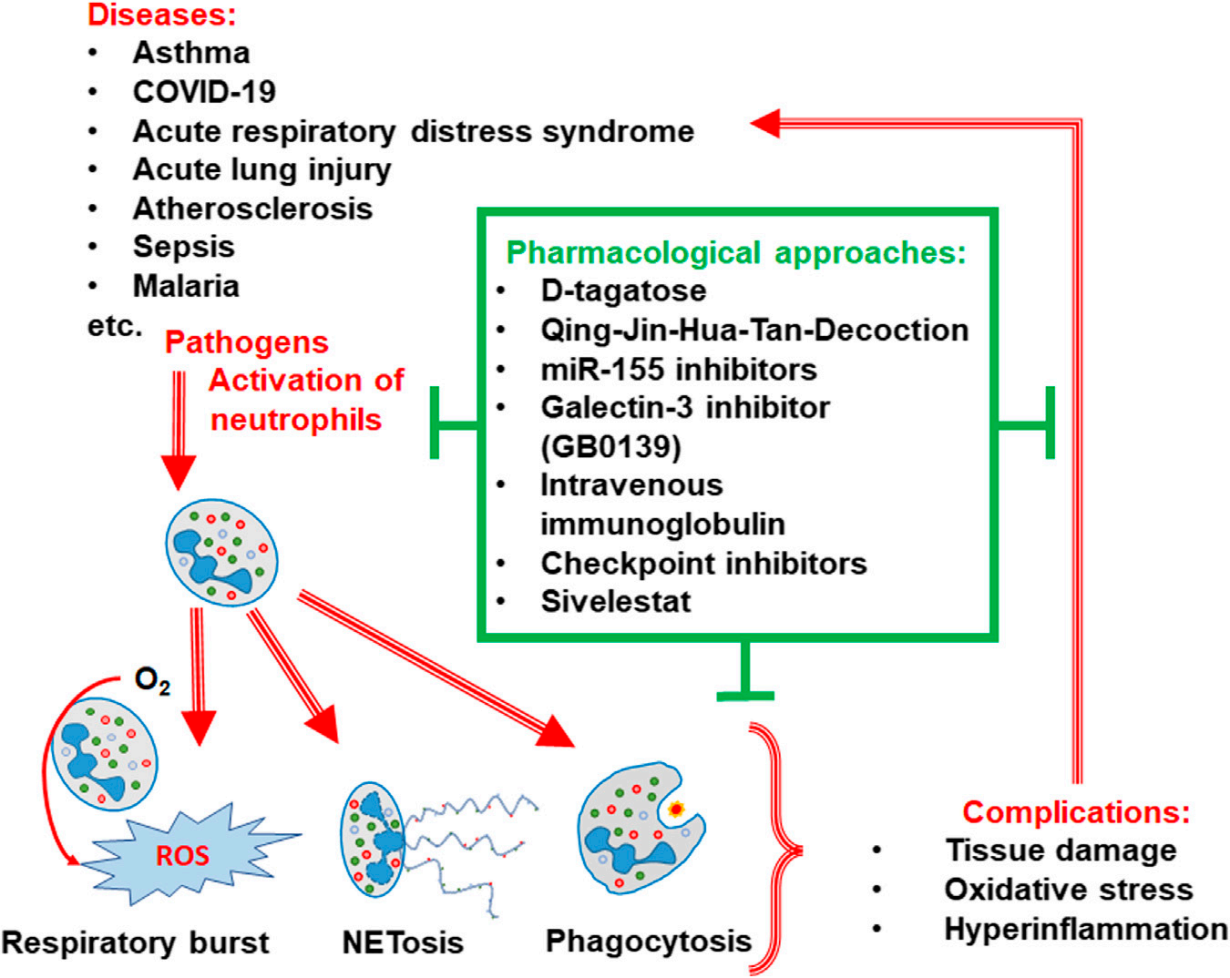
Pharmacological approaches targeting neutrophilic inflammation.

In summary, the papers in this topic issue illustrate the involvement of neutrophils in various pathologies and the role of neutrophils in host immunity (Scheme 1).
